# Impact of exercise training on myocardial contractile functions assessed by cardiac magnetic resonance in post-myocardial infarction patients

**DOI:** 10.1186/s43044-022-00288-4

**Published:** 2022-06-23

**Authors:** Ahmed Elshazly, Rana Lateef Hasan, Mohamed Ayman Saleh, Ahmed Samir Ibrahim, Hazem Khorshid

**Affiliations:** Department of Cardiology, Ain Shams University, Cairo, Egypt

**Keywords:** Myocardial infarction, Cardiac magnetic resonance, Exercise training, Cardiac rehabilitation

## Abstract

**Background:**

Improvement of functional capacity and mortality reduction in post-MI patients were found to be associated with regular exercise training. The cardiac magnetic resonance (CMR) is considered the most accurate non-invasive modality in quantitative assessment of left ventricular (LV) volumes and systolic functions. Our main objective was to investigate the impact of exercise training on LV systolic functions in patients post anterior MI using CMR. 32 patients on recommended medical treatment 4 week after having a successful primary PCI for an anterior MI were recruited, between May 2018 and May 2019. They were divided into two groups, training group (TG): 16 assigned to a 12 week exercise training program and control group (CG): 16 who received medical treatment without participating in the exercise training program. Treadmill exercise using modified Bruce protocol was done to TG before and after the training program in order to record the resting and maximum HR, metabolic equivalent (MET), and calculate HR reserve. CMR was performed for all patients 4 weeks after PCI and was repeated after completion of the study period to calculate ejection fraction (EF), left ventricular end-systolic volume (LVESV), left ventricular end-diastolic volume (LVEDV), and wall motion score index (WMSI).

**Results:**

100% were males. 6 patients from CG dropped during follow-up, no statistically significant difference between the two groups regarding age, BMI, smoking status, hypertension, diabetes mellitus and dyslipidemia. Using the CMR, the TG showed significant improvement in EF (36.6 ± 14.2% to 43.1 ± 12.4%; *P* < 0.001) and WMSI (2.03 ± 0.57 to 1.7 ± 0.49; *P* < 0.001), without statistically significant change in LV volumes. Regarding CG no significant changes in EF, WMSI, LV volumes were found. There was significant improvement in EF and WMSI change before and after study in TG vs. CG [6.5 (2.3–9.0) vs. − 2.0 (− 6.8 to 1.3), *P* value < 0.001] and [− 0.3 (− 0.5 to 0.1) vs. 0.1 (− 0.1 to − 0.5), *P* value 0.001] respectively.

**Conclusions:**

12 weeks of exercise training program in post-MI patients have a favorable impact on LV global and regional systolic functions without adversely affecting LV remodeling (as assessed by CMR).

## Background

In post myocardial infarction (MI) patients, regular exercise training was associated with improvement of functional capacity, control of cardiovascular risk factors, and reduction of mortality [1]. Some studies showed improvement in functional capacity and even reduction in LV volumes in post-MI patients with either preserved EF, moderately or severely reduced EF after planned exercise training program [2–4]. Non-invasive assessment of LV volumes and systolic functions can be done with different imaging modalities; the cardiac magnetic resonance (CMR) is considered the most accurate modality in comparison to other imaging modalities [3].

Assessment of the LV volumes, global and regional systolic functions using CMR is much more accurate and reproducible than using 2D echocardiography, with no risk of cumulative hazardous radiation exposure during frequent follow-up [5]. One of the strong independent mortality predictors in post-MI patients was found to be wall motion score index (WMSI) which can be evaluated accurately using CMR [6, 7].

Ischemic heart diseases in most cases affect regional areas of the myocardium. This makes measure of global function insensitive to changes in regional performance, in many cases with ischemic heart disease normal EF can conceal underlying significant regional abnormalities. Moreover, since the heart during different phases of cardiac cycle moves through and rotates within any imaging plane, tomographic imaging of endocardial and epicardial motion has a complex relationship [6].

Anatomical images provided by CMR show clearly the inner borders of endocardium and outer borders of epicardium of the heart chambers, which provide valuable data regarding LV global and regional functions [6].

Hence, the aim of the present study was to investigate the impact of exercise training on LV systolic functions (global and regional) in patients post anterior MI using CMR.

## Methods

The study included 32 patients on optimal medical treatment 4 weeks after having a successful primary PCI for anterior MI referred to our hospital cardiac rehabilitation (CR) unit between May 2018 and May 2019. The patients were randomized into two groups: training group (TG) *n* = 16 assigned to a 12 weeks exercise training program and control group (CG): 16 (six patients dropped during follow-up) who received standard guideline-directed medical treatment without participating in exercise training program. Stratified randomization was used to ensure equal numbers in the different categories of LV EF (LV EF < 20%, 20–50, and > 50%).

Exclusion criteria included: patients with metallic heart valves, claustrophobia, history of CABG or previous PCI, those with remaining significant coronary lesions after the primary PCI, patients with implanted cardiac pacemaker except for MRI-safe pacing system, patients with a metallic foreign body, Patient’s refusal, decompensated heart failure, uncontrolled arrhythmia, advanced heart block, musculoskeletal disease, severe systemic illness and severe valvular disease. All patients were subjected to full history and thorough physical examination.

Patients on beta blockers, Renin Angiotensin Aldosteron System (RAAS) inhibiting drugs and other heart failure disease modifying treatment continued using same doses throughout the study.

Both groups were subjected to formal CR program including risk factor assessment and control, psychosocial support, nutritional and physical activity counseling. Calculation of BSA was done using the following equation: Body surface area = Square root of [Height (cm) × Weight (kg)/3600] [8], in order to calculate LV volumes indexed to BSA.

Treadmill exercise test using modified Bruce protocol (symptom-limited) was done on all participants in the TG before and after the training program in order to record the resting and maximum HR, metabolic equivalent (MET) and calculate HR reserve (maximum HR-resting HR).

Before participating in the training program, the program was explained to all participants and informed written consent was obtained. Training program done by TG was scheduled for 3 times every week, each session was designed as a moderate intensity treadmill training to achieve 40–60% of the heart rate reserve, calculated from the baseline treadmill exercise test using modified Bruce protocol (symptom-limited) done before joining the training program. Duration of each session was 30 min preceded by 5 min warm-up training such as stretching and walking. Supervision during treadmill exercise was performed by nurse or one of our study team. In order to monitor the progression of exercise intensity, Borg scale of rate of perceived exertion (RPE) was used. In absence of symptoms, the patients were exercised at 11–13 RPE. Patient monitoring included heart rate, blood pressure, continuous ECG and rating of perceived exertion (RPE) pre and post-exercise session.

Non-contrast Cardiac Magnetic Resonance (CMR) using 1.5-Tesla Philips (Philips Healthcare Systems), a phased array cardiac coil of 16 channels was performed for all patients 4 weeks after PCI and was repeated after completion of the study period that lasted for 12 weeks to calculate ejection fraction (EF), left ventricular end-systolic volume (LVESV), left ventricular end-diastolic volume (LVEDV) and wall motion score index (WMSI). CMR imaging was performed with a cardiac coil positioned over the heart. Cardiac gating of the sequences was obtained by applying 4 magnetic resonance compatible electrocardiographic electrodes. Using a steady-state free precession pulse sequence (SSFP), cine images were recorded after obtaining axial, coronal and sagittal images. Short axis cine imaging of the entire left ventricle, from the base to the apex, was performed for the analysis of cardiac volumes and function. Number of slices acquired was 9–15 slices with slice thickness of 8 mm. Each cardiac phase has an acquisition window of 80–100 ms with temporal resolution of 40–50 ms. Assessment of wall motion score index (WMSI) was done according to the 16-segment model (Six basal segments, six mid segments, and four apical segments) [9].

Each segment is then scored, using the following criteria: 1 for normal thickening, 2 for hypokinesia, 3 for akinesia, and 4 for dyskinesia. The calculation of WMSI was done by dividing the sum of the calculated segmental scores by the number of segments. A WMSI of 1.0 (16/16) is considered normal. The image analysis was performed offline by a single observer, with approved post possessing workstation Philips Health Care System version 5.

The collected data were coded, tabulated, and statistically analyzed using IBM SPSS statistics (Statistical Package for Social Sciences) software version 22.0, IBM Corp., Chicago, USA, 2013. Quantitative normally distributed data described as mean ± SD (standard deviation) after testing for normality using Shapiro–Wilk test, then compared using independent *t*-test (two independent groups) and paired t-test (paired data), if not normally distributed described as Median (1st–3rd Interquartiles) then compared using Mann Whitney test (two independent groups) and Wicoxon signed rank (paired data). Qualitative data described as number and percentage and compared using Chi square test and Fisher’s Exact test for variables with small expected numbers. The level of significance was taken at *P* value < 0.050 was significant, otherwise was non-significant.

## Results

Thirty-two patients with anterior MI were enrolled in the present study 4 weeks after successful primary PCI. The patients were randomly assigned to control group (CG) *n* = 16 (six patients dropped during follow-up) who received standard guideline-directed medical treatment without participating in exercise training program and exercise training group (TG) *n* = 16 who received standard guideline-directed medical treatment as well as joining exercise training program. 100% of the study group were males, mean age was 50.1 ± 8.6 years in training group vs. 51.1 ± 9.6 years in control group (*P* value 0.95), mean BMI was 28.1 ± 2.9 kg/m^2^ in training group vs. 27.4 ± 3.1 kg/m^2^ in control group (*P* value 0.61), no statistically significant difference between the two groups regarding smoking status, hypertension, diabetes mellitus and dyslipidemia (*P* values: 0.99, 0.99, 0.36 and 0.99 respectively).

Demographic data and risk factors for both groups are shown in Table 1. Patients on beta blockers, RAAS inhibiting drugs, and other heart failure disease modifying treatment continued using the same doses during the study. The medical treatment of both groups is shown in Table 2.

**Table 1:** Demographic data and risk factors for both groups

Demographic data	Training group	Control group	*P* value
Total number	16	10	
Gender			
Male	16 (100.0%)	10 (100.0%)	Not applicable
Age (years)	50.1 ± 8.6	51.1 ± 9.6	0.95^
BMI (Body Mass Index)	28.1 ± 2.9 kg/m^2^	27.4 ± 3.1 kg/m^2^	0.61^
NYHA classification			
NYHA I	7 (44%)	6 (60%)	0.51^#^
NYHA II	4 (25%)	3 (30%)	
NYHA III	5 (31%)	1 (10%)	
Baseline data			
Ex-smoker	10 (62%)	7 (70%)	0.99^#^
Current smoker	4 (25%)	2 (20%)	0.99^#^
HTN	7 (44%)	4 (40%)	0.99^#^
DM	3 (19%)	4 (40%)	0.36^#^
Dyslipidemia	7 (44%)	5 (50%)	0.99^#^

^Independent *t* test. ^#^Fisher’s Exact test

**Table 2:** Medical treatment of both groups

Drugs name	Training group (*n* = 16)	Control group (*n* = 10)	*P* value^#^
Antiplatelet			
Aspirin tablet 75 mg	16 (100%)	10 (100%)	Not applicable
Clopidogrel tablet 75 mg	9 (56%)	6 (60%)	0.99^#^
Ticagrelor tablet 90 mg	7 (43%)	4 (40%)	0.99^#^
Nitrate tablet	2 (12%)	1(10)	0.99^#^
Beta-blocker	16 (100%)	10 (100%)	Not applicable
ACEI and ARBs	16 (100%)	10 (100%)	Not applicable
Statin	16 (100%)	10 (100%)	Not applicable
Loop diuretics	4 (25%)	2 (20%)	0.99^#^
Mineralocortico Receptor Antagonists (MRAs)	4 (25%)	2 (20%)	0.99^#^

^#^Fisher’s Exact test

There was a statistically significant increase in heart rate reserve after exercise training program (69.2 ± 13.5 bpm) as compared to the baseline before (63.8 ± 12.1 bpm) (p 0.021). Mean resting HR decreased in a statistically significant way after exercise training (68.3 ± 5.4 bpm) as compared to baseline (76.2 ± 8.6 bpm) (*P* < 0.001). Moreover, Functional capacity significantly improved after the exercise program as evidenced by a statistically significant increase in METs (*P* < 0.001) achieved by the patients during modified Bruce exercise after the training program (8.1 ± 2.1) as compared to the baseline before (5.3 ± 2.1).

Regarding maximum HR and resting systolic blood pressure both showed no significant changes (140.1 ± 16.1–137.6 ± 15.1, *P* value 0.15) and (112.1 ± 14.4–111.5 ± 13.3, *P* value 0.89), however, there was a statistically significant reduction in resting diastolic BP (75.2 ± 8.3–66.6 ± 7.8, *P* 0.004).

Heart rate (resting, maximum and reserve), METs, resting blood pressures (systolic and diastolic) of the TG before and after the training program are shown in Table 3.

**Table 3:** Heart rate (resting, maximum and reserve), METs, resting blood pressures (systolic and diastolic) of the TG before and after the training program

	Before exercise training program	After exercise training program	Mean Diff	*P* value^
Resting Heart rate (bpm)	76.2 ± 8.6	68.3 ± 5.4	− 7.875	< 0.001*
Maximum Heart rate (bpm)	140.1 ± 16.1	137.6 ± 15.1	− 2.5	0.15
Heart rate reserve (maximum HR-Resting HR)	63.8 ± 12.1	69.2 ± 13.5	5.37	0.021*
Metabolic equivalent (METs)	5.3 ± 2.1	8.1 ± 2.1	2.75	< 0.001*
Resting systolic BP	112.1 ± 14.4	111.5 ± 13.3	− 0.625	0.89
Resting diastolic BP	75.2 ± 8.3	66.6 ± 7.8	− 8.625	0.004*

^Paired *t* test, *Significant

In addition, LVEF, WMSI, Left Ventricular End Diastolic Volume (LVEDV), Left Ventricular End Systolic Volume (LVESV) and LV volumes indexed to BSA of both groups were measured by CMR as shown in Table 4.

**Table 4:** CMR parameters (EF, WMSI, LVESV, LVEDV, and LV volumes indexed to BSA) before and after the study

Variables	Time	Training group (*n* = 16)	Control group (*n* = 10)	*P* value^
EF (%)	Before	36.6 ± 14.2	41.0 ± 10.6	0.412^
	After	43.1 ± 12.4	37.2 ± 11.4	0.229^
	Change	6.5 (2.3–9.0)	− 2.0 (− 6.8 to 1.3)	0.001*^,$^
	*P* value#	< 0.001*	0.212	
WMSI	Before	2.03 ± 0.57	1.8 ± 0.4	0.220
	After	1.7 ± 0.49	1.9 ± 0.5	0.264
	Change	− 0.3 (− 0.5 to − 0.1)	0.1 (− 0.1 to 0.5)	0.001*^,$^
	*P* value#	< 0.001*	0.331	
LVESV (mL)	Before	79.6 ± 39.8	82.2 ± 28.0	0.858^
	After	72.6 ± 35.0	89.9 ± 35.4	0.233^
	Change	− 1.5 (− 25.5 to 9.7)	11.4 (− 13.8 to 28.3)	0.135^**$**^
	*P* value#	0.255	0.44	
LVEDV (mL)	Before	119.9 ± 40.8	135.8 ± 39.6	0.336
	After	122.8 ± 43.1	139.7 ± 34.4	0.306
	Change	17.6 (− 37.6 to 36.8)	2.5 (− 37.0 to 44.8)	0.816
	*P* value#	0.918	0.64	
LVESV indexed to BSA ( mL/m2)	Before	40.4 ± 19.8	41.4 ± 13.9	0.894
	After	36.7 ± 16.6	45.4 ± 18.6	0.228
	Change	− 0.5 (− 14.0 to 5.3)	5.1 (− 6.9 to 13.8)	0.150^**$**^
	*P* value#	0.215	0.386	
LVEDV indexed to BSA ( mL/m^2^)	Before	61.2 ± 20.5	67.9 ± 16.9	0.393
	After	62.2 ± 19.1	70.6 ± 18.6	0.281
	Change	9.1 (− 20.3 to 15.6)	1.5 (− 18.1 to 23.4)	0.660^**$**^
	*P* value#	0.836	0.575	

Before and after Data presented as Mean ± SD, while change data presented as Median (1st − 3rd IQ). ^Independent *t* test. ^**$**^Mann Whitney test. #Wilcoxon signed rank test. *Significant

Although there was no significant difference in LVEDV and LVESV mean values either indexed to BSA or not before and after completion of the study in training group, there was a statistically significant increase in LVEF (36.6 ± 14.2%–43.1 ± 12.4%, *P* < 0.001) and statistically significant improvement in WMSI (2.03 ± 0.57–1.7 ± 0.49, *P* < 0.001) as assessed by CMR. There was no statistically significant difference in the LVEDV and LVESV mean values in control group (135.8 ± 39.6 mL to 139.7 ± 34.4 mL, *P* value 0.64), (82.2 ± 28.0 mL to 89.9 ± 35.4 mL, *P* value 0.44) respectively, the same was found when the LV volumes were indexed to BSA. Moreover, there was no statistically significant difference neither in WMSI mean values (1.8 ± 0.4 to 1.9 ± 0.5, *P* value 0.33) nor in EF mean value (41.0 ± 10.6% to 37.2 ± 11.4%, *P* value 0.21) in control group as compared to baseline.

Regarding change in CMR parameters between TG and CG before and after the study, there was statistically significant improvement regarding EF change in TG [6.5 (2.3–9.0) vs. − 2.0 (− 6.8 to 1.3), *P* value < 0.001], significant improvement of WMSI change [− 0.3 (− 0.5 to 0.1) vs. 0.1 (− 0.1 to − 0.5), *P* value 0.001].

**Fig. 1:**
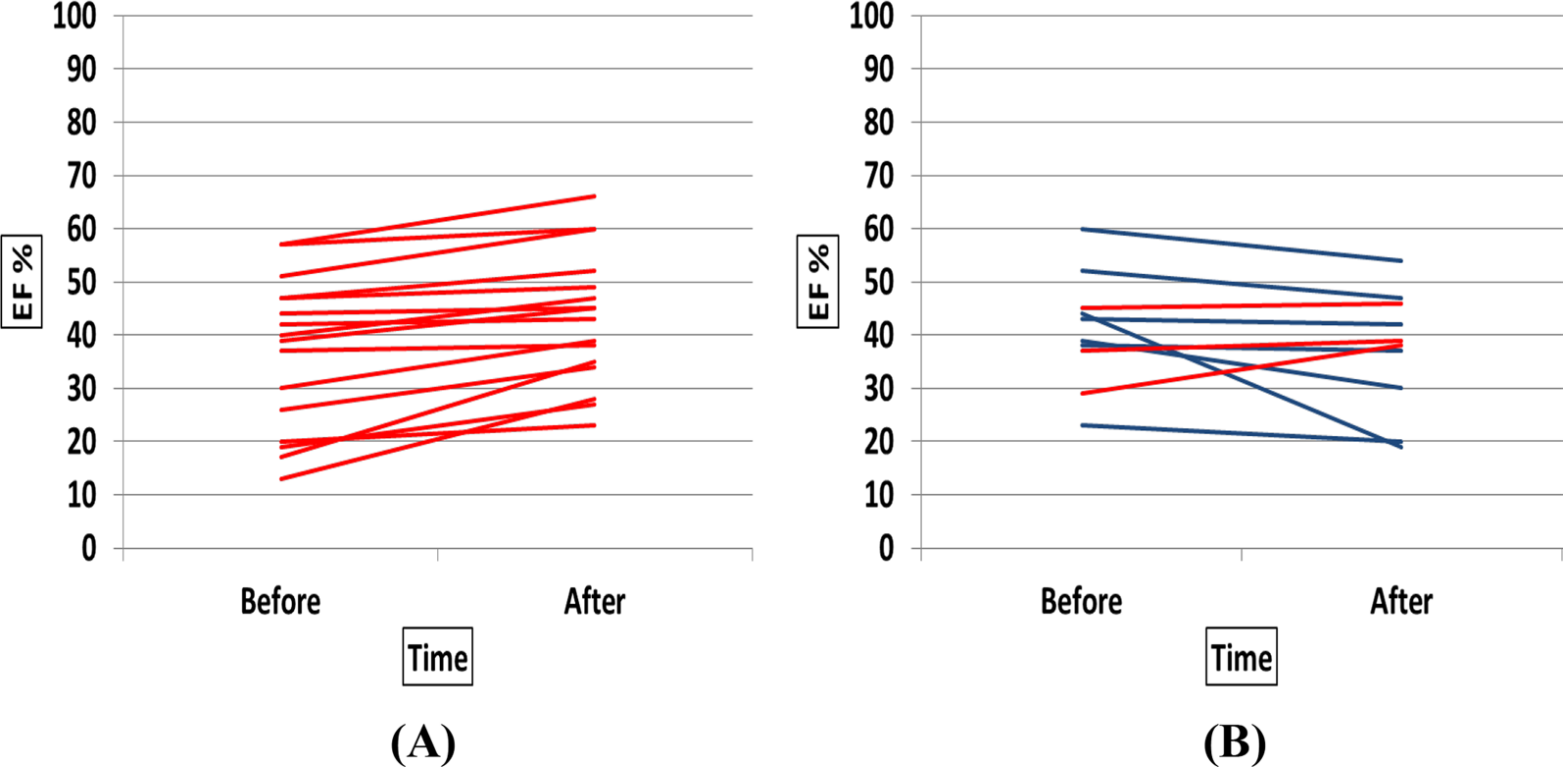
EF in individual patients of TG (**A**) vs. CG (**B**) before and after the study (Red lines were for cases with improved EF)

**Fig. 2:**
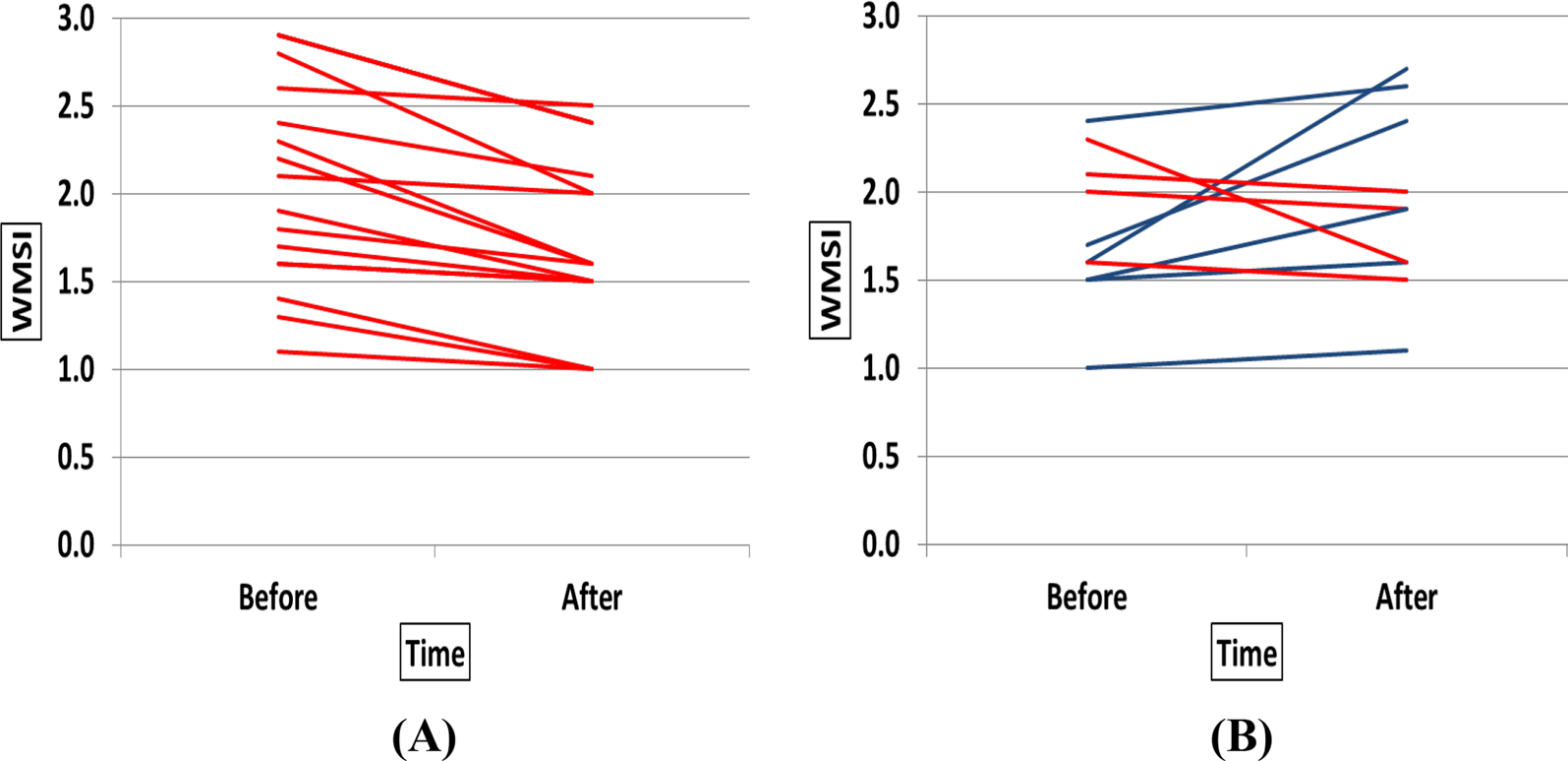
WMSI in individual patients of TG (**A**) vs. CG (**B**) before and after the study (Red lines were for cases with improved WMSI)

Changes in EF and WMSI in individual patients of TG vs. CG before and after the study are shown in Figs. 1 and 2.

**Fig. 3:**
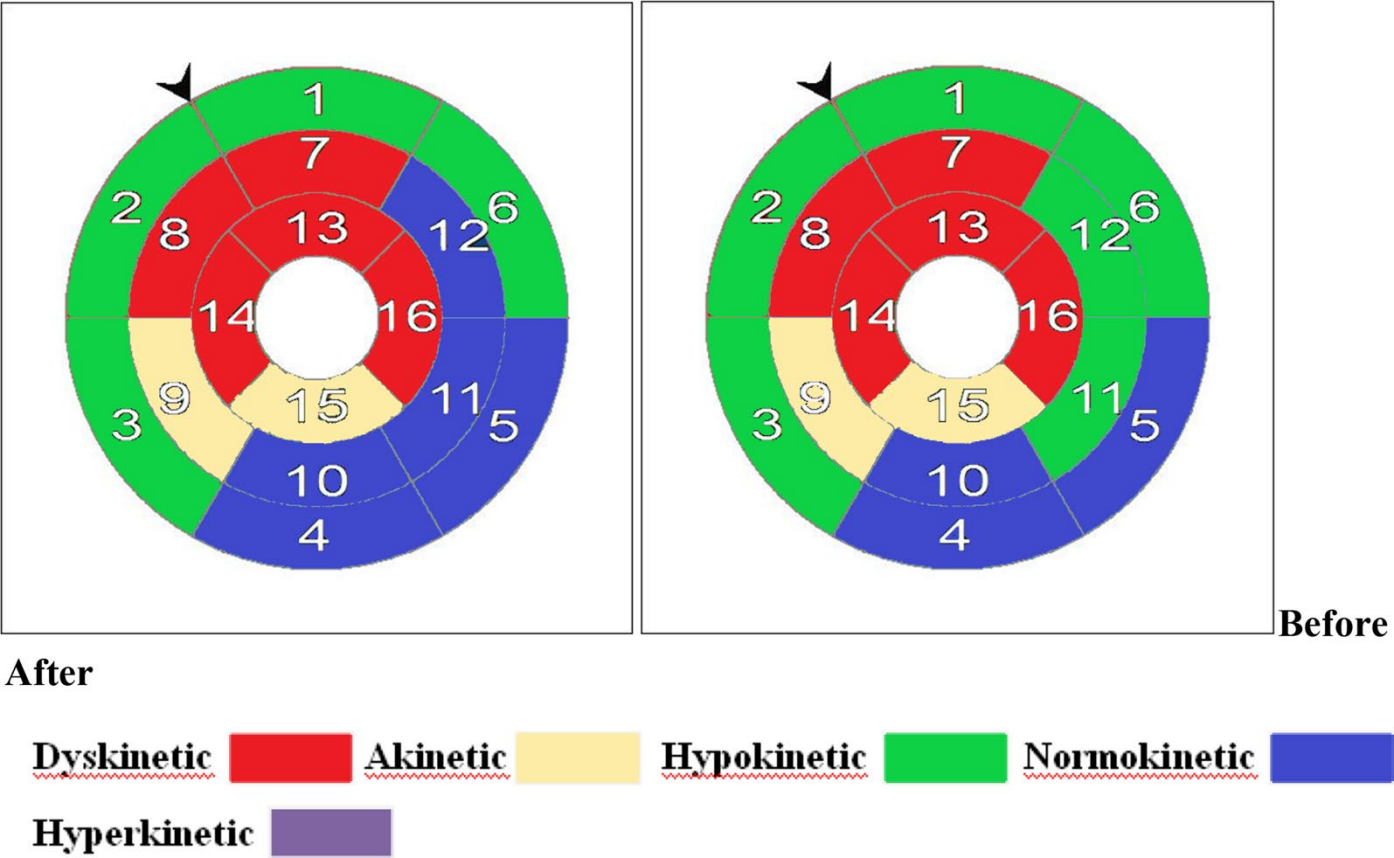
Patient No. 1 (CG): 59-year-old exsmoker, hypertensive patient with established diagnosis of anterior STEMI underwent primary PCI then enrolled to the CG. By the end of the study there was a deterioration in EF (23 vs.20) % and WMSI (2.4 VS. 2.6) shown by American heart association (AHA) CMR Bull’s eye plot segmental WMS of the left ventricle

**Fig. 4:**
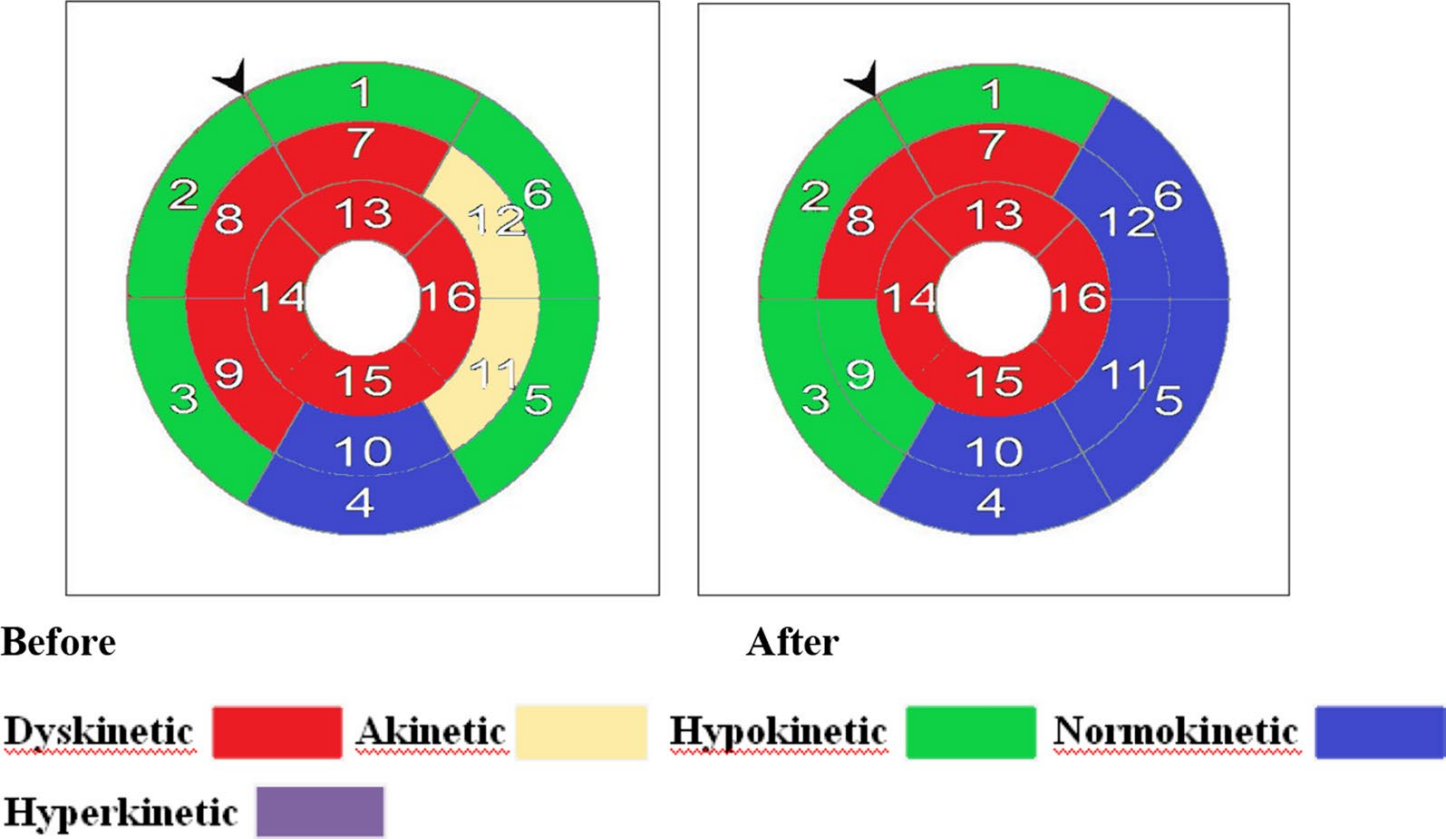
Patient No. 10 (TG): 43-year-old male patient, smoker with established diagnosis of anterior STEMI, underwent successful primary PCI then enrolled to the TG. By the end of the training program there was an improvement of EF (19 vs. 27) % and WMSI (2.9 VS. 2.4) shown by American heart association (AHA) CMR Bull’s eye plot segmental WMS of the left ventricle

Demonstration of American heart association (AHA) LV 16 segments Bull’s eye plot segmental WMS by CMR for a patient from each group (TG and CG) before and after the study is represented in Figs. 3 and 4.

## Discussion

By using CMR, the present study showed that 12 weeks of exercise-based CR in patients post-MI resulted in a statistically significant increase in LVEF and significant improvement of WMSI, but there was no significant difference in LVEDV and LVESV mean values before and after the end of the training program, significant reductions in LV volumes might have happened with earlier initiation and longer duration of exercise training program [10]. On the other hand, the control group showed no statistically significant difference in LVEF, WMSI, LVEDV or LVESV.

Our findings were in line with some of the previous studies regarding effect of exercise on LV EF. [4, 11–15]

Giallauria et al. randomized 61 post myocardial infarction (MI) patients to CG 31 and TG 30 patients. Exercise program for the TG was designed for 6 months. The assessment of LV volumes and functions for the whole study group before and after the study period was done using echocardiography. They found that trained patients showed an improvement in workload (26% increase, *P* < 0.001), ejection fraction (EF), LV end-diastolic volume index (LVEDVI; reduction by 9%, *P* < 0.001) and NT-proBNP [11].

In another study Giallauria et al. randomized 46 recent STEMI patients to TG (25) and CG (21), TG was subjected to 6 months exercise-based CR program. dipyridamole myocardial perfusion single photon emission computed tomography was done on the whole study group within first week after the MI and after 6 months. They found significant improvement in TG when compared to CG in resting and post- dipyridamole stress wall motion score indexes (*P* < 0.01 in both) and resting and post- dipyridamole stress LV ejection fraction (*P* < 0.05 in both) [12].

Haddadzadeh et al. enrolled 42 patients within one month after acute coronary syndrome, the study group was randomized to 3 months training program TG (center-based exercise training 19 patients and home-based exercise training 9 patients) and CG (14 patients) with no exercise training. Significant improvement in EF was found in the TG when compared to CG, while no difference were found between center based and home-based exercise training [13].

On the other hand, Dubach et al. using CMR concluded that no difference was observed in EF, LVEDV and LVESV with substantial increases in exercise capacity. 25 patients post-MI or CABG (15% of the study group were post CABG patients) were included in that study and were divided into training group (mean age 56 ± 5 years, mean ejection fraction 32 ± 7%, *n* = 12) and control group (mean age 55 ± 7 years, mean EF 33 ± 6%, *n* = 13). Exercise program was performed over 2 months duration and it was a high-intensity training program consisting of daily walking for 2 h and 4 sessions a week of high-intensity stationary cycling (40 min at 70–80% peak capacity). [16]

In another CMR study conducted by Myers et al. on twenty-five post-MI patients with impaired LV systolic function who underwent bypass surgery. and were randomly assigned to training group (*n* = 12, aged 56 ± 5 years, ejection fraction 31.5% ± 7%) and control group (control group: *n* = 13, aged 55 ± 7 years, ejection fraction 33.3% ± 6%). Exercise program was performed over 2 months duration and it was a high-intensity training program (was quantified by number of blocks walked or flights of stairs climbed per day). Measures of left ventricular volumes and function were assessed by CMR before exercise program, 2 month after starting the program and 1 year later. No significant changes were found in LV volumes or ejection fraction in either training or control groups throughout the study period [17].

De Santi et al. also studied the effect of exercise training in sixteen patients with anterior wall myocardial infarction who were randomized into two groups: training (*n* = 8) and control (*n* = 8), seven patients were not eligible for any revascularization strategy at time of admission and were kept only on medical treatment without revascularization. Seven patients received fibrinolytic therapy and only two patients were treated with primary percutaneous coronary intervention. The training program was moderate-intensity aerobic training. CMR was done to all participants as a baseline and after 12 weeks. It was observed that there was no significant changes in LVEF, LVEDV and LVESV mean values in both control and training group [18].

Discrepancies among those several studies could be attributed to factors related to heterogeneity of samples [16], differences in training intensity [16, 17], diversity of the strategies used in the management of ischemia [17, 18], or even a combination of these factors.

## Conclusions

The present study showed that 3 sessions a week of moderate intensity treadmill exercise training for 12 weeks in post-MI patients have a favorable impact on LV global and regional systolic functions without adversely affecting LV remodeling (as assessed by CMR) and was associated with significant improvement in exercise capacity.

### Limitations of the study

1. Relatively short follow-up duration.
2. Patients drop during follow-up.
3. Another limitation was the exclusion of 5 patients due to claustrophobia or inability to hold breathing during CMR study.
4. LV strain analysis was not done.
5. Study of scar size was not done as intravenous contrast was not given.

## Data Availability

The datasets used and analyzed during the current study are available from the corresponding author on reasonable request.

## Abbreviations

CMR: Cardiac magnetic resonance
WMSI: Wall motion score index
TG: Training group
CG: Control group
BSA: Body surface area
CR: Cardiac rehabilitation
PCI: Percutaneous coronary intervention
RAAS: Rennin angiotensin aldosterone system
MET: Metabolic equivalent
RPE: Rate of perceived exertion
HRR: Heart rate reserve
